# A latent class approach to identify multi‐risk profiles associated with phylogenetic clustering of recent hepatitis C virus infection in Australia and New Zealand from 2004 to 2015

**DOI:** 10.1002/jia2.25222

**Published:** 2019-02-12

**Authors:** Sofia R Bartlett, Tanya L Applegate, Brendan P Jacka, Marianne Martinello, Francois MJ Lamoury, Mark Danta, Daniel Bradshaw, David Shaw, Andrew R Lloyd, Margaret Hellard, Gregory J Dore, Gail V Matthews, Jason Grebely

**Affiliations:** ^1^ Kirby Institute UNSW Sydney NSW Australia; ^2^ St Vincent's Clinical School UNSW Sydney NSW Australia; ^3^ Department of Gastroenterology St Vincent's Hospital Sydney Sydney Australia; ^4^ National Infection Service Public Health England London UK; ^5^ Royal Adelaide Hospital Adelaide SA Australia; ^6^ School of Medical Sciences UNSW Sydney NSW Australia; ^7^ The Burnet Institute Melbourne Vic. Australia

**Keywords:** human immunodeficiency virus, hepatitis C virus, co‐infection, phylogenetic clustering, latent class analysis, multi‐risk profiles, gay and bisexual men, people who inject drugs

## Abstract

**Introduction:**

Over the last two decades, the incidence of hepatitis C virus (HCV) co‐infection among men who have sex with men (MSM) living with HIV began increasing in post‐industrialized countries. Little is known about transmission of acute or recent HCV, in particular among MSM living with HIV co‐infection, which creates uncertainty about potential for reinfection after HCV treatment. Using phylogenetic methods, clinical, epidemiological and molecular data can be combined to better understand transmission patterns. These insights may help identify strategies to reduce reinfection risk, enhancing effectiveness of HCV treatment as prevention strategies. The aim of this study was to identify multi‐risk profiles and factors associated with phylogenetic pairs and clusters among people with recent HCV infection.

**Methods:**

Data and specimens from five studies of recent HCV in Australia and New Zealand (2004 to 2015) were used. HCV Core‐E2 sequences were used to infer maximum likelihood trees. Clusters were identified using 90% bootstrap and 5% genetic distance threshold. Multivariate logistic regression and latent class analyses were performed.

**Results:**

Among 237 participants with Core‐E2 sequences, 47% were in a pair/cluster. Among HIV/HCV co‐infected participants, 60% (74/123) were in a pair/cluster, compared to 30% (34/114) with HCV mono‐infection (*p *< 0.001). HIV/HCV co‐infection (vs. HCV mono‐infection; adjusted odds ratio (AOR), 2.37, 95% confidence interval (CI), 1.45, 5.15) was independently associated with phylogenetic clustering. Latent class analysis identified three distinct risk profiles: (1) people who inject drugs, (2) HIV‐positive gay and bisexual men (GBM) with low probability of injecting drug use (IDU) and (3) GBM with IDU & sexual risk behaviour. Class 2 (vs. Class 1, AOR 3.40; 95% CI, 1.52, 7.60), was independently associated with phylogenetic clustering. Many clusters displayed homogeneous characteristics, such as containing individuals exclusively from one city, individuals all with HIV/HCV co‐infection or individuals sharing the same route of acquisition of HCV.

**Conclusions:**

Clusters containing individuals with specific characteristics suggest that HCV transmission occurs through discrete networks, particularly among HIV/HCV co‐infected individuals. The greater proportion of clustering found among HIV/HCV co‐infected participants highlights the need to provide broad direct‐acting antiviral access encouraging rapid uptake in this population and ongoing monitoring of the phylogeny.

## 1 Introduction

Globally, the prevalence and incidence of hepatitis C virus (HCV) infection among people who inject drugs (PWID) is high, with approximately 42.4% to 62.1% of PWID estimated to be HCV antibody positive 1. The prevalence and incidence of HCV infection among human immunodeficiency virus (HIV)‐positive gay and bisexual men (GBM) is also considerable, with prevalence estimated to be between 5.3% and 7.3% 2, 3, 4, 5, 6, 7. While variations exist in the incidence and prevalence of HCV infection among HIV‐positive GBM across geographical regions, transmission of HCV has been sustained among this population in recent years 8, 9. Ongoing and overlapping transmission of HCV among these groups highlights the need for further investigation of factors that influence transmission of this virus 10, 11. While it is hypothesized that treatment as prevention strategies using direct‐acting antiviral (DAA) therapies may contribute to HCV elimination 12, 13, 14, 15, 16, 17, 18, more detailed characterization of the transmission of HCV is needed to guide the implementation of these strategies 19, 20.

Beginning in the late 1990s, the incidence of HCV co‐infection in HIV‐positive GBM began to increase in high‐income countries 3, 21, such as Switzerland 22 and the United Kingdom 23. The incidence of HCV infection in these populations remains high to the present time 9. The findings were mirrored in Australia, with specific transmission networks identified among HIV‐positive GBM 5, 24. A model including both sexual and drug use risk behaviour 25, 26, 27 was proposed to explain HCV transmission among HIV‐positive GBM, highlighting the complex nature of transmission. Phylogenetic studies of recent HCV infection found that HIV co‐infection and HCV genotype 1a were associated with transmission clusters 28, 29.

Phylogenetic analyses can uncover patterns of disease transmission 30, 31, rather than just patterns of disease acquisition, such as in traditional epidemiological studies. While phylogenetic techniques cannot determine the exact direction of transmission, sources and trends can be identified on a population level 32, 33. By combining data from these analyses with detailed behavioural, clinical and demographic data, underlying networks can be detected, that may otherwise remain hidden 34, 35.

Latent class analysis (LCA) has been used to characterize patterns of polydrug use and other types of multi‐risk profiles in relation to HIV and HCV acquisition 36, 37, 38. However, it has only recently been combined with phylogenetic data to understand transmission risk for HIV and HCV 39, 40. LCA assumes the population consists of sub‐populations (latent classes) that differ in their distributions of included variables and provides the ability to identify these latent classes. The ability to stratify analyses based on HIV infection status with increased study size, and insights provided by LCA, combined with phylogenetic analysis, delivers a unique opportunity to better understand transmission of HCV among different groups. These insights could identify potential targets for the optimal implementation of treatment as prevention and provide a foundation for the future evaluation of the effectiveness of treatment as prevention.

The aim of this study was to identify multi‐risk profiles and factors associated with phylogenetic clustering of recent HCV infection in Australia and New Zealand between 2004 and 2015 among people with and without HIV infection.

## 2 Methods

### 2.1 Study population and design

Data and specimens from five studies of recent HCV (duration of infection <18 months) in Australia and New Zealand were used for this study: ATAHC 5, RAMPT‐C 41, ATAHC II/DARE‐C I 42 and DARE‐C II 43. Participants were recruited through a network of tertiary clinics and hospitals between 2004 and 2015 (published elsewhere 5, 41, 42, 43 and described in Data S1). For inclusion in this study, participants had to have recent HCV defined as initial detection of serum anti‐HCV antibody and/or HCV RNA within six months of enrolment and either (i) documented recent HCV seroconversion (anti‐HCV antibody negative result in the 18 (DARE‐C II) or 24 (ATAHC, ATAHC II, DARE‐C I, RAMPT‐C) months prior to enrolment) or (ii) acute clinical hepatitis (jaundice or alanine aminotransferase (ALT) greater than 10 times the upper limit of normal (ULN)) within the previous 12 months with exclusion of other causes of acute hepatitis, and estimated duration of HCV infection <12 (DARE‐C II) or 18 (ATAHC, ATAHC II, DARE‐C I, RAMPT‐C) months at screening. Calculation of the estimated date of infection for subjects is described in Data S1. The first available HCV RNA‐positive Ethylenediaminetetraacetic acid or acid‐citrate‐dextrose plasma sample following detection of HCV was selected. All participants provided a written informed consent and protocols were approved by appropriate Human Research Ethics Committees.

### 2.2 HCV RNA sequencing and phylogenetic analysis

HCV RNA was extracted, Core‐E2 region amplified (nucleotides 347 to 1750 in H77 reference sequence (GenBank accession no. NC_004102)), then Sanger sequenced (method published elsewhere 44 and described in Data S1). The fragment analysed was 1104 bp long following removal of hypervariable region one (HVR1) to improve cluster resolution 44. Sequences were aligned using ClustalW 45 with reference sequences from the Los Alamos National Laboratory HCV database 46 and unrelated sequences from overseas 47, 48 to disrupt spurious clustering and support identification of locally expanding of clusters 49. Maximum likelihood phylogenetic trees were inferred for genotypes 1, 3 and 2/4/6 combined in RAxML 50 through CIPRES Science Gateway 51 under the general time reversible model of nucleotide substitution with substitution rate heterogeneity and 1000 bootstrap replicates. JModelTest 52, 53 was used to determine the nucleotide substitution model. Clusters and pairs were identified using ClusterPicker 54 with 90% bootstrap support threshold and 5% mean maximum genetic distance cutoff. Sensitivity analyses, performed by varying genetic distance threshold between 1.5% and 5% with and without 90% bootstrap threshold, and previous studies 28, 44, determined 5% mean maximum genetic distance was the most epidemiologically relevant cutoff to define clustering for this population.

### 2.3 Study outcomes

The primary study outcome was phylogenetic clustering of HCV infections, as defined by two or more participants with HCV genome sequence within the bootstrap and genetic distance threshold cutoff. A pair was defined as two participants within the cutoff and a cluster was defined as three or more participants within the cutoff.

### 2.4 Latent class analysis

LCA was used to identify groups of participants sharing behavioural and epidemiological characteristics, to identify multi‐risk profiles associated with phylogenetic clustering 39. LCA models were built using only risk behaviour and basic demographic variables to enhance real‐world applicability of resulting multi‐risk profiles. The LCA model included all available variables indicating risk behaviours related to HCV transmission; mode of HCV acquisition (sexual acquisition or injecting drug use (IDU) acquisition, defined by clinician), IDU (never injected, injected but not within the last six months or injected within the last six months and the last drug that was injected) 55, 56, 57, 58, sex and older age (in categories: <45, >45 years). Multiple models were estimated with varying numbers of classes (from one to eight classes) and no covariate in SAS (version 9.4: Sas Institute Inc., Cary, NC, USA), using the PROC LCA plugin 59, 60. Bayesian information criterion (BIC), Akaike information criterion (AIC), adjusted BIC (aBIC) and adjusted AIC (aAIC) were used to determine the best‐fitting model, in addition to entropy and epidemiological meaningfulness of class structure. The best‐fitting model was run with distal outcome (phylogenetic clustering) and each participant had posterior probability of belonging to each latent class of the fitted model calculated. For subsequent analysis 39, 61, participants were allocated to the latent class for which they had the highest posterior membership probability, with class treated as an observed variable in adjusted logistic regression analysis.

### 2.5 Statistical analyses

Multivariate logistic regression analysis was used to identify multi‐risk profiles and factors associated with being in a pair or cluster. Factors hypothesized to be associated with being in a pair or cluster that were assessed included: older age 5, 62, 63, male sex (vs. female sex) 64, HIV infection or sexual acquisition of HCV 5, 6, 7, 65 and recent injection drug use (defined as injecting anytime in the last six months prior to screening) 12, 66, 67. Due to collinearity between HCV/HIV co‐infection and sexual acquisition of HCV (all persons with clinician assigned sexual acquisition were HCV/HIV co‐infected), models were constructed adjusting for these factors separately. Analyses were also stratified by HIV infection status, and to account for potential unmeasured confounding introduced by cohort characteristics, adjusted logistic regression analysis was performed using mixed modelling, with a random intercept for cohort. For all analyses, statistically significant differences were assessed at *p *< 0.05; *p*‐values are two‐sided. All analyses were performed using STATA software (version 14; StataCorp L.P., College Station, TX, USA).

## 3 Results

### 3.1 Study population

**Figure 1:**
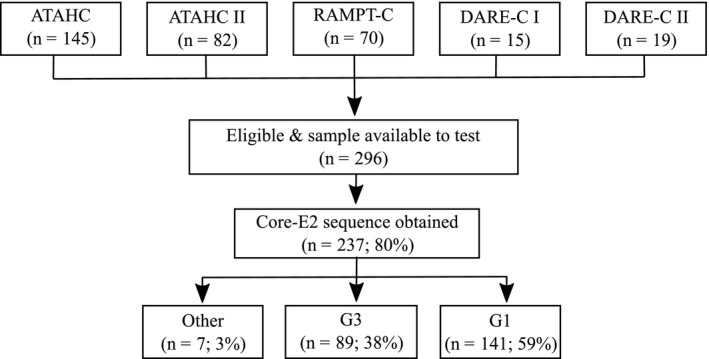
Flow chart of sources of participants and sequences from five studies of recent hepatitis C virus (HCV) infection in Australia between 2004 and 2015 ATAHC, Australian Trial in Acute Hepatitis C; RAMPT‐C, Defining risk and mechanisms of permucosal transmission for acute HCV infection within high‐risk populations; ATAHC II, Australian Trial in Acute Hepatitis C II; DARE‐C I, DAA‐based therapy for recently acquired hepatitis C I; DARE‐C II, DAA‐based therapy for recently acquired hepatitis C II; E2, envelope 2; G, genotype.

In total, 296 subjects were eligible for inclusion in this study (Figure 1), with 237 HCV Core‐E2 sequences obtained. The characteristics of participants with a Core‐E2 sequence are shown in Table 1. The median age was 37 (interquartile range 29 to 46) years, 79% were male, 84% were White people and 52% were HIV positive. Homosexual exposure was universally reported as a risk factor for HIV acquisition among those with HCV/HIV co‐infection (n = 123).

**Table 1:** Characteristics of participants with an available hepatitis C virus (HCV) Core‐E2 sequence from five studies of recent HCV infection in Australia and New Zealand recruited between 2004 and 2015

Characteristic	Overall	ATAHC I	RAMPT‐C	ATAHC II	DARE‐C I	DARE‐C II
Period of study recruitment/follow‐up		2004 to 2007	2009 to 2013	2011 to 2013	2013 to 2015	2014 to 2015
Period of study recruitment/follow‐up		2004 to 2007	2009 to 2013	2011 to 2013	2013 to 2015	2014 to 2015
Total n (%)	(n = 237)	(n = 119)	(n = 25)	(n = 60)	(n = 15)	(n = 18)
Age (median years, Q2 to Q3)	38 (29 to 46)	33 (25 to 41)	45 (37 to 50)	41 (32 to 47)	46 (44 to 53)	44 (31 to 50)
Gender						
Female	37 (16%)	28 (24%)	a	8 (13%)	0 (0%)	1 (6%)
Male	187 (79%)	81 (68%)	25 (100%)	52 (87%)	13 (87%)	16 (89%)
Other^b^	13 (5%)	10 (8%)	0 (0%)	0 (0%)	2 (13%)	1 (6%)
City						
Sydney	109 (46%)	46 (39%)	14 (56%)	28 (47%)	13 (87%)	8 (44%)
Melbourne	88 (37%)	50 (42%)	11 (44%)	22 (37%)	0 (0%)	5 (28%)
Adelaide	27 (11%)	15 (13%)	a	10 (17%)	2 (11%)	a
Other^c^	13 (6%)	8 (7%)	a	a	a	5 (28%)
HIV infection						
Positive	123 (52%)	36 (30%)	24 (96%)	38 (63%)	11 (73%)	14 (78%)
Negative	114 (48%)	83 (70%)	1 (4%)	22 (37%)	4 (27%)	4 (22%)
Acquisition of HCV^d^						
Sexual	97 (41%)	36 (30%)	18 (72%)	26 (43%)	9 (60%)	8 (44%)
Injecting drug use	121 (51%)	68 (57%)	7 (28%)	33 (55%)	4 (27%)	9 (50%)
Unknown	19 (8%)	15 (13%)	0 (0%)	1 (2%)	2 (13%)	1 (6%)
Estimated year of HCV acquisition						
2003 to 2005	72 (30%)	72 (%)	a	a	a	a
2006 to 2008	48 (20%)	47 (%)	1 (4%)	a	a	a
2009 to 2011	43 (19%)	a	22 (88%)	20 (33%)	1 (7%)	a
2012 to 2014	74 (31%)	a	2 (8%)	40 (67%)	14 (93%)	18 (100%)
HCV genotype						
1a	131 (55%)	59 (50%)	17 (68%)	30 (50%)	14 (93%)	11 (61%)
1b	10 (4%)	8 (7%)	0 (0%)	1 (2%)	1 (7%)	0 (0%)
3a	89 (38%)	48 (40%)	7 (28%)	28 (46%)	0 (0%)	6 (33%)
2/4/6	7 (3%)	3 (3%)	1 (4%)	1 (2%)	0 (0%)	1 (6%)
Injection drug use						
Never injected	57 (24%)	18 (15%)	14 (56%)	15 (25%)	7 (47%)	3 (16%)
Injected ever, but not recently^e^	78 (33%)	52 (44%)	5 (20%)	10 (17%)	2 (13%)	9 (50%)
Injected recently^e^	89 (37%)	42 (35%)	6 (24%)	34 (56%)	2 (13%)	5 (28%)
Unknown	13 (5%)	7 (6%)	0 (0%)	1 (2%)	4 (27%)	1 (6%)
Drug recently^e^ injected^f^						
Heroin	16 (18%)	14 (33%)	0 (0%)	2 (6%)	0 (0%)	0 (0%)
Methadone/buprenorphine	18 (20%)	18 (43%)	0 (0%)	0 (0%)	0 (0%)	0 (0%)
Other opioids	7 (8%)	5 (12%)	0 (0%)	1 (3%)	1 (50%)	0 (0%)
Methamphetamine/amphetamine	30 (34%)	0 (0%)	6 (100%)	18 (53%)	1(50%)	5 (100%)
Unknown	18 (20%)	5 (12%)	0 (0%)	13 (38%)	0 (0%)	0 (0%)
Opioid substitution therapy ever	25 (11%)	16 (13%)	0 (0%)	8 (13%)	1 (7%)	0 (0%)

Percentages indicate column percentages, except for drug last injected^f^.

ATAHC, Australian Trial in Acute Hepatitis C; RAMPT‐C, Defining risk and mechanisms of permucosal transmission for acute HCV infection within high‐risk populations; ATAHC II, Australian Trial in Acute Hepatitis C II; DARE‐C I, DAA‐based therapy for recently acquired hepatitis C I; DARE‐C II, DAA‐based therapy for recently acquired hepatitis C II; Q, quartiles; NA, variable not available for study.

^a^Variable not applicable to study; ^b^other includes one transgender subject and 12 subjects for which variable was unavailable; ^c^Newcastle, Brisbane, Auckland or Perth; ^d^acquisition was determined by the clinician according to reported risk factors; ^e^within last six months prior to sample date; ^f^among people who reported recent injecting (within last six months prior to sample date).

### 3.2 Phylogenetic pair and cluster composition

**Figure 2:**
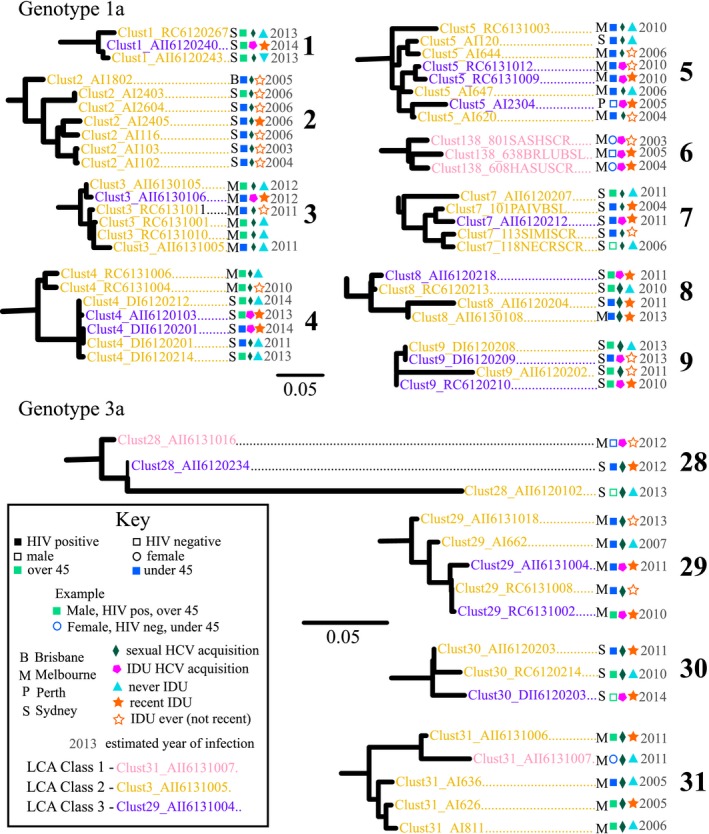
Clusters from maximum likelihood phylogenetic trees, constructed with sequences from Core‐E2 region of hepatitis C virus (HCV) obtained from people with recent infection in Australia between 2004 and 2015 (full trees in Figure S1) All identified clusters at <5% mean maximum genetic distance cutoff are displayed (genotype 1a numbered #1 to 9 and genotype 3a numbered #28 to 31). Scale bars indicate nucleotide substitutions per site. Tip names are coloured by latent class analysis (LCA) highest posterior probability classes (Class 1: PWID; Class 2: HIV‐positive GBSM or Class 3: GBSM with injecting drug use (IDU)). Numbers at tips represent estimated year of infection for each participant (if available) and letters represent the city where participants were recruited. Squares represent males, circles females, filled circles or squares represent a participant with HCV/HIV co‐infection, empty circles or squares represent HCV mono‐infection, and light green represents participants who are over 45 years of age, with blue representing under 45 years of age. Small diamonds represent participants who acquired HCV infection sexually, with pentagons representing IDU acquisition. A triangle represents participants never reporting IDU, an empty star represents reporting IDU ever but not recently and a filled star represents reporting recent IDU.

Phylogenetic trees were constructed separately for genotypes 1, 3 and G2/4/6 combined (Figure S1). Overall, 46% of participants were in a pair or cluster, with 60% (74/123) of HCV/HIV co‐infected participants in a pair or cluster compared to 30% (34/114) of HCV mono‐infected participants (*p *< 0.001). Clusters ranged in size from three to eight participants, shown in Figure 2. Many clusters displayed homogeneous characteristics, such as clusters containing exclusively HCV/HIV co‐infected individuals (Clusters 1 to 4, 8, 9, 29, Figure 2), individuals with sexual acquisition of HCV infection (Clusters 2 and 31, Figure 2) or IDU acquisition (Cluster 6, Figure 2), individuals with history of IDU (Clusters 2 and 6, Figure 2) or individuals from one city (Clusters 1, 3, 6, 7, 9, 29 to 31, Figure 2). Some clusters displayed heterogeneous characteristics, such as mixing of age categories, route of acquisition of HCV and IDU history.

### 3.3 Factors associated with membership in a pair or cluster overall

In a logistic regression model adjusting for age, gender and city, only HIV infection remained associated with membership in a pair/cluster (vs. HCV mono‐infection; adjusted odds ratio (AOR), 2.30; 95% confidence interval (CI), 1.07, 4.94) (Table S1). In a logistic regression model adjusting for age, gender, city and mode of HCV acquisition, only HCV genotype 3a infection (vs. genotype 1a; AOR, 2.09, 95% CI, 1.11, 3.95) and infection with an HCV genotype other than 1a or 3a (vs. genotype 1a; AOR, 3.98, 95% CI, 1.21, 13.02) remained associated with being in a pair/cluster (Table S1).

### 3.4 Factors associated with membership of a pair or cluster, stratified by HIV infection status

In logistic regression analysis stratified by HIV infection status, among HCV mono‐infected participants, only HCV genotype 3a (vs. genotype 1a; AOR, 4.35, 95% CI, 1.42, 13.30) was associated with being in a pair/cluster (Table S2). Among HCV/HIV co‐infected participants, no factors were significant (Table S3).

### 3.5 Multi‐risk profiles

After comparison of fit statistics, a model with three classes was found to be best fit (Table S4). Based on item response probabilities for observed classes, multi‐risk profiles were named according to relative distributions of participant characteristics (Table 2). Class 1 was named “PWID,” as class probability for having recently injected drugs or acquiring HCV through IDU were highest for this class, and no participants assigned to this class had HIV infection. Class 2 was named “HIV‐positive GBM with low probability of IDU,” as class probability for being male was almost 1, probability of acquiring HCV sexually was almost 1, probability of having never injected drugs was highest in this class, and almost all participants assigned to this class had HIV co‐ infection. Class 3 was named “GBM with IDU & sexual risk behaviour,” as class probability for being male was almost 1, probability of recently injecting methamphetamine was highest, and the majority of participants had HIV co‐ infection. Almost all clusters contained mostly participants assigned to Class 2, with small numbers of participants assigned to Class 3 distributed among these clusters. Only three clusters contained participants assigned to Class 1, with this class having the lowest likelihood of being in a cluster.

**Table 2:** Response probability for characteristics of the three multi‐risk profiles identified by Latent Class Analysis among five studies of recent hepatitis C virus (HCV) infection in Australia and New Zealand recruited between 2004 and 2015

Characteristic	Class response probability		
	Class 1	Class 2	Class 3
	PWID	HIV‐positive GBM with low probability of IDU	GBM with IDU & sexual risk behaviour
Probability of class membership	0.31	0.39	0.30
Aged over 45 years	<0.01	0.52	0.33
Male	0.53	0.96	0.98
Acquisition of HCV^b^			
IDU	>0.99	<0.01	0.79
Sexual	<0.01	>0.99	0.21
IDU history			
Most recently^^^ injected heroin	0.31	0.01	<0.01
Most recently^^^ injected methamphetamine^a^	0.29	0.11	0.52
Have injected ever, but not recently	0.38	0.25	0.48
Never injected	0.02	0.63	<0.01
HIV positive^c^	<0.01	0.96	0.60

HCV, hepatitis C virus; PWID, people who inject drugs; GBM, gay and bisexual men; IDU, injecting drug use; HIV, human immunodeficiency virus.

^recent defined as within last 6 months; ^a^Methamphetamine or amphetamine; ^b^acquisition was determined by the clinician according to reported risk factors; ^c^HIV co‐infection was not included in model used to build latent classes due to collinearity with sexual acquisition of HCV. However, proportion of people with HIV co‐infection in each class was estimated here by assigning individuals to the class with highest posterior membership probability.

### 3.6 Multi‐risk profiles associated with being in a pair or cluster

In unadjusted logistic regression analysis, both Class 2 “HIV‐positive GBM with low probability of IDU’’ and Class 3 “GBM with IDU & sexual risk behaviour” (vs. Class 1 PWID) were associated with membership in a pair/cluster (Table 3). In adjusted analysis, membership in a pair/cluster was associated with Class 2 (vs. Class 1; AOR, 3.40, 95% CI, 1.52, 7.60), HCV genotype 3a infection (vs. genotype 1a; AOR, 1.94, 95% CI, 1.06, 3.57) and infection with a non 1a/3a HCV genotype (vs. genotype 1a; AOR, 4.26, 95% CI, 1.31, 13.84).

**Table 3:** Multivariate logistic regression of factors associated with phylogenetic clustering, including multi‐risk profiles, among hepatitis C virus (HCV) Core‐E2 sequences (at 5% genetic distance threshold) among participants from five studies of recent HCV infection in Australia and New Zealand recruited between 2004 and 2015

Characteristic	Overall	Unclustered	Clustered	Membership in cluster n ≥ 2								
Total n (%)	(n = 237)	(n = 129)	(n = 108)	Unadjusted			Adjusted for HIV infection			Adjusted for multi‐risk profile		
				Odds ratio	95% CI	*p*	Odds ratio	95% CI	*p*	Odds ratio	95% CI	*p*
City												
Other^a^	40 (17%)	28 (21%)	12 (11%)	Ref	–	–	Ref	–	–	Ref	–	–
Sydney	109 (46%)	57 (44%)	52 (48%)	2.13	0.98, 4.61	0.056	1.15	0.47, 2.81	0.753	1.41	0.60, 3.29	0.433
Melbourne	88 (37%)	44 (34%)	44 (41%)	2.33	1.05, 5.17	0.037	1.71	0.73, 4.02	0.217	2.18	0.93, 5.09	0.072
HIV infection												
Negative	114 (48%)	80 (62%)	34 (31%)	Ref	–	–	Ref	–	–	NI	NI	NI
Positive	123 (52%)	49 (38%)	74 (69%)	3.55	2.07, 6.09	<0.001	**2.73**	**1.45, 5.15**	**0.002**	NI	NI	NI
HCV genotype												
1a	131 (55%)	86 (67%)	45 (42%)	Ref	–	–	Ref	–	–	Ref	–	–
3a	89 (38%)	38 (29%)	51 (47%)	2.56	1.47, 4.46	0.001	1.83	0.99, 3.37	0.052	**1.94**	**1.06, 3.57**	**0.032**
Other	17 (7%)	5 (4%)	12 (11%)	4.59	1.52, 13.83	0.007	**3.28**	**1.02, 10.54**	**0.046**	**4.26**	**1.31, 13.84**	**0.016**
Multi‐risk profile^b^												
Class 1 PWID	59 (25%)	45 (34%)	14 (13%)	Ref	–	–	NI	NI	NI	Ref	–	–
Class 2 HIV‐positive GBM with low probability of IDU	97 (41%)	42 (33%)	55 (51%)	4.21	2.05, 8.66	<0.001	NI	NI	NI	**3.40**	**1.52, 7.60**	**0.003**
Class 3 GBM with IDU & sexual risk behaviour	81 (34%)	42 (33%)	39 (36%)	2.98	1.42, 6.26	0.004	NI	NI	NI	2.22	0.96, 5.15	0.062

Percentages indicate column percentages. Factors remaining significant in adjusted analyses (*p* < 0.05) are highlighted in bold.

HIV, human immunodeficiency virus; HCV, hepatitis C virus; PWID, people who inject drugs; GBM, gay and bisexual men; IDU, injecting drug use, CI, confidence interval; NI, not included; Ref, reference.

^a^Adelaide, Newcastle, Auckland, Brisbane or Perth; ^b^multi‐risk profile assigned corresponds to the profile with the highest posterior probability for that individual.

## 4 Discussion

This study characterizes associations between overlapping and co‐occurring risk factors and HCV phylogenetic clustering among participants from five studies of recent HCV infection in Australia and New Zealand between 2004 and 2015. HIV/HCV co‐infection, recruitment in Melbourne and HCV genotype 3a infection were independently associated with being in a pair or cluster. LCA identified three multi‐risk profiles that included: (1) “PWID”, (2) “HIV‐positive GBM with low probability of IDU” and (3) “GBM with IDU & sexual risk behaviour.” Phylogenetic clustering was independently associated with membership in risk profile (2) “HIV‐positive GBM with low probability of IDU” after adjusting for other factors. These findings suggest that there are different sub‐populations at risk of HCV transmission even within those identifying as having a sexual or drug use risk. Thus, although both risk groups 2 and 3 had potential for sexual transmission, networks were able to be potentially identified based on combinations of risk factors. Different strategies may be warranted to address transmission within different networks. These findings identify a combination of participant characteristics that may be associated with HCV transmission or acquisition, providing potential targets for the implementation of public health interventions. This study describes a robust methodology for understanding populations at greater risk of viral transmission where risk factors overlap or co‐occur.

The association between HCV subtype 3a and phylogenetic clustering, with all clusters containing individuals infected over multiple years, is consistent with other reports of an increased proportion of incident HCV infection as a result of subtype 3a, compared to 1a, particularly among HIV‐negative PWID 68, a smaller population of infected people, and more recent introduction of subtype 3a to Australia, compared to 1a 69. This phenomenon has also been observed in countries such as Scotland 70, Germany 71, 72, England 73, Canada and the United States 69. This contrasts with a previous analysis which found an association between HCV subtype 1a and phylogenetic clustering 28, which may be explained by the more recent period of recruitment and higher proportion of participants with HCV/HIV co‐infection sampled in this study. This observed recent increase in transmission of subtype 3a supports broad availability and uptake of potent pan‐genotypic DAA regimens.

This study found that HCV/HIV co‐infection was independently associated with phylogenetic clustering. HIV infection was acquired exclusively homosexually among participants with HCV/HIV co‐infection in this study; however, many participants with HCV/HIV co‐infection reported both sexual and drug risk factors for HCV acquisition. While evidence has emerged that supports sexual transmission of HCV among GBM, both with and without HIV co‐infection 41, 74, 75, the presence of co‐occurring and overlapping risk factors among participants may conceal the contribution that sexual networks have on HCV transmission. While sexual acquisition of HCV infection was not associated with phylogenetic clustering, membership in the multi‐risk profile Class (2) “HIV‐positive GBM with low probability of IDU” was independently associated with phylogenetic clustering. This multi‐risk profile consisted of males who exclusively had HCV/HIV co‐infection, acquired HCV infection sexually and reported very little IDU, either recently or ever. This pattern was also evident in clusters observed that contained HIV‐positive men with no history of IDU and reported sexual acquisition of HCV (e.g. Clusters 3 and 31, Figure 2). This supports previous findings suggesting the sexual networks among HIV‐positive GBM through which HCV is transmitted are highly connected in Australia 24, and have potentially been densely sampled in this study, particularly compared to injecting networks among heterosexual PWID. It is also possible that IDU is under‐reported in this population, due to stigma associated with it 26, 76, 77, particularly in healthcare settings such as where these studies were recruited from.

The diagnosis of acute HCV infection has recently increased among HIV‐negative GBM 78, 79, 80. While this may be driven by increased testing and heightened awareness of HCV infection risk among this population, it has raised concern that with increased uptake of pre‐exposure prophylaxis (PrEP) to prevent HIV infection 81, 82, HCV infections may continue to rise among HIV‐negative GBM. It is possible that real time detection of this type of phylogenetic signal could be useful as a trigger to implement more in depth public health monitoring and interventions, such as increasing awareness around risk of sexual transmission of HCV among GBM 83, 84, and tailoring education to individuals based on their HIV infection status 85. Phylogenetic analysis of HCV NS5B sequences from HIV‐negative GBM receiving PrEP in Amsterdam demonstrated GBM‐specific HCV clusters containing both HIV‐positive and HIV‐negative individuals 86. Interventions implemented because of real time detection of phylogenetic signals in HCV are being developed and evaluated in the Netherlands and the United States 87, and may be useful in Australia to reduce transmission of HCV and investigate HCV outbreaks.

The multi‐risk profile Class (3) “GBM with IDU & sexual risk behaviour” had a combination of HCV acquisition through both sexual and drug use, and reported high proportions of recent methamphetamine injection, indicating the overlapping concurrent transmission risks present. Membership in this group was not independently associated with phylogenetic clustering. This finding suggests that members were more likely to have acquired their infection from people who were not sampled in this study, and that these networks are both broader and have not been sampled densely in this study. Those not sampled in this study were people with chronic HCV infection, and potentially people who are less likely to attend tertiary clinics or hospitals where participants in these studies were recruited. People who may be less likely to attend such settings are marginalized people or those not engaged in the healthcare system, particularly PWID 88, 89. This highlights the need to provide HCV testing and treatment in non‐tertiary clinics and other places where the people who need to access these services are most likely to visit. This also suggests that different strategies to prevent and treat HCV infection among GBM who inject methamphetamine may be needed to reduce transmission of HCV infection in this group.

This study demonstrates that LCA can be extremely useful to identify critical differences in potential transmission risk between groups that remain otherwise hidden. The methods described here can be used to examine unmeasured subgroups of participants based on multiple indicators, rather than individual factors, and overcomes some of the difficulties with traditional epidemiological methods used to investigate risk factors. While the classes identified do not represent actual individuals in the population, the LCA provides a useful mechanism for representing the heterogeneity of factors across the population.

Limitations include limited sampling of extremely high‐risk populations, such as PWID, particularly those in prison or otherwise unengaged in tertiary care, and the exclusion of chronically infected individuals. The network through which HCV is transmitted among HIV‐positive GBM has been sampled densely, in comparison to the network through which HCV is transmitted among HIV‐negative PWID. This is likely to have influenced the high overall proportion of phylogenetic clustering observed in this study. There is also difficulty in distinguishing between sexual and IDU as the route of HCV infection acquisition among people who report both categories of risk factors. However, creating multi‐risk profiles as done in this analysis can help to overcome this issue. There were also sampling bias in the way people were recruited to these studies, as they were conducted in tertiary care settings, and without any network‐based or respondent‐driven recruitment. Sampling was also limited by geographical area, with only selected sites in a limited number of Australian and New Zealand cities recruiting subjects; therefore, this study is not a random sample of the eligible populations and contains some bias.

## 5 Conclusions

A high proportion of phylogenetic clustering observed among participants with HCV/HIV co‐infection suggests transmission of HCV may occur through highly connected networks of HIV‐positive GBM. Increased screening and rapid delivery of HCV DAA treatment as prevention among HIV‐positive GBM should be considered, as it may be effective to reduce transmission of HCV in this population. There may also be a role for real time monitoring of the phylogeny, to detect signals related to transmission “hot spots” and trigger implementation of public health interventions. Transmission of HCV and HIV can occur rapidly through injecting and sexual networks 90, 91, and outbreak investigation using phylogenetic clustering analyses could improve monitoring and detection of emerging epidemics. This study provides a foundation upon which transmission of HCV among people with recent infection can be evaluated in the future, particularly in the setting of implementation of treatment as prevention to eliminate HCV infection among particular populations.

## Competing interest

Dr. Grebely is a consultant/advisor and has received research grants from AbbVie, Bristol Myers Squibb (BMS), Cepheid, Gilead Sciences and Merck. Dr. Dore is a consultant/advisor and has received research grants from Abbvie, BMS, Gilead, Merck, Janssen and Roche. Dr. Martinello has received speaker payments from Abbvie. Dr. Hellard and Dr. Lloyd received investigator initiated research funding from Gilead Sciences, Abbvie and BMS. Dr. Bradshaw has received investigator imitated research funding from Viiv and Janssen.

## Authors’ contributions

GVM was the principal investigator of the ATAHC II, DARE‐C I and DARE‐C II studies. MD was the principal investigator of the RAMPT‐C study. GJD, MH and DS were the co‐investigators for the ATAHC, ATAHC II, DARE‐C I, DARE‐C II and RAMPT‐C studies. SRB, TLA, GJD, GVM and JG conceived and designed this study, with input from BPJ, MM, MD, DB, ARL and MH. SRB, FMJL and DB performed all the laboratory work. SRB had access to the data in the study and takes responsibility for the integrity of the data and the accuracy of the results. SRB performed the statistical analyses with input from MM, BPJ, JG, GVM and TLA. SRB wrote the first draft of the article with input from JG, GVM, GJD and TLA. All authors critically reviewed the first draft of the article and approved the final version to be submitted.

## Supporting information


**Data S1.** Supplementary Materials and Methods.
**Figure S1.** Maximum likelihood phylogenetic trees inferred from available hepatitis C virus (HCV) Core‐E2 sequence from five studies of recent HCV infection in Australia and New Zealand recruited between 2004 and 2015.
**Table S1.** Multivariate logistic regression of factors associated with phylogenetic clustering among hepatitis C virus (HCV) Core‐E2 sequences (at 5% genetic distance threshold) among participants from five studies of recent HCV infection in Australia and New Zealand recruited between 2004 and 2015
**Table S2.** Multivariate logistic regression of factors associated with phylogenetic clustering among hepatitis C virus (HCV) Core‐E2 sequences (at 5% genetic distance threshold) stratified among **HCV mono‐infected** participants from five studies of recent HCV infection in Australia and New Zealand recruited between 2004 and 2015
**Table S3.** Multivariate logistic regression of factors associated with phylogenetic clustering of hepatitis C virus (HCV) Core‐E2 sequences (at 5% genetic distance threshold) among **HIV/HCV co‐infected** participants from five studies of recent HCV infection in Australia and New Zealand recruited between 2004 and 2015
**Table S4.** Comparison of fit statistics for latent class analysis models built with 1 to 8 classes for participants from five studies of recent HCV infection in Australia and New Zealand recruited between 2004 and 2015
