# Supplementary material for: A latent class approach to identify multi‐risk profiles associated with phylogenetic clustering of recent hepatitis C virus infection in Australia and New Zealand from 2004 to 2015
Source: J Int AIDS Soc. 2019 Feb 12;22(2):e25222. doi: 10.1002/jia2.25222 (PMC6371014; doi:10.1002/jia2.25222)
Supplement: Supplementary file 1 — Data S1. Supplementary Materials and Methods. Figure S1. Maximum likelihood phylogenetic trees inferred from available hepatitis C virus (HCV) Core‐E2 sequence from five studies of recent HCV infection in Australia and New Zealand recruited between 2004 and 2015. Table S1. Multivariate logistic regression of factors associated with phylogenetic clustering among hepatitis C virus (HCV) Core‐E2 sequences (at 5% genetic distance threshold) among participants from five studies of recent HCV infection in Australia and New Zealand recruited between 2004 and 2015 Table S2. Multivariate logistic regression of factors associated with phylogenetic clustering among hepatitis C virus (HCV) Core‐E2 sequences (at 5% genetic distance threshold) stratified among HCV mono‐infected participants from five studies of recent HCV infection in Australia and New Zealand recruited between 2004 and 2015 Table S3. Multivariate logistic regression of factors associated with phylogenetic clustering of hepatitis C virus (HCV) Core‐E2 sequences (at 5% genetic distance threshold) among HIV/HCV co‐infected participants from five studies of recent HCV infection in Australia and New Zealand recruited between 2004 and 2015 Table S4. Comparison of fit statistics for latent class analysis models built with 1 to 8 classes for participants from five studies of recent HCV infection in Australia and New Zealand recruited between 2004 and 2015 [file JIA2-22-e25222-s001.docx]

**Supplementary Materials and Methods**

*Study population*

The Australian Trial in Acute Hepatitis C (ATAHC) was a prospective, multicentre longitudinal study of natural history and treatment outcomes of recent HCV infection in Australia that recruited 145 participants at Australian sites between 2004 and 2007 [[1](#_ENREF_1)]. The Defining risk and mechanisms of permucosal transmission for acute HCV infection within high-risk populations (RAMPT-C) study was a longitudinal cohort study of HCV RNA positive men not currently receiving therapy for HCV, which recruited 70 men from the hepatitis clinic at St Vincent’s Hospital, Sydney between 2009 and 2013 [[2](#_ENREF_2)]. The Australian Trial in Acute Hepatitis C II (ATAHC II) study was a prospective, multicentre longitudinal study of natural history and treatment outcomes following response guided treatment of recent hepatitis C infection that recruited 82 participant at Australian sites between 2011 and 2015 [[3](#_ENREF_3)]. The DAA based therapy for recently acquired hepatitis C I (DARE-C I) study was a multicentre open label study on the effectiveness of response guided triple therapy with PEG-IFN, ribavirin and telaprevir in patients infected with HCV genotype 1 that recruited 14 participants from Australian sites between 2013 and 2015 [[3](#_ENREF_3)]. The DAA based therapy for recently acquired hepatitis C II (DARE-C II) study was a multicentre open label single arm study on the effectiveness of sofosbuvir and ribavirin for treatment of patients with estimated duration of infection ≤12 months with all HCV genotypes that recruited 19 participants from Australian and New Zealand sites between 2014 and 2015 [[4](#_ENREF_4)].

*Estimated date of infection*

The estimated date of clinical HCV infection was calculated as six weeks before the onset of seroconversion illness or six weeks before the first alanine aminotransferase (ALT) greater than ten times the upper limit of normal (ULN). The estimated date of asymptomatic HCV infection was calculated as the midpoint between the last negative anti-HCV antibody or HCV RNA and the first positive anti-HCV antibody or HCV RNA. For participants who were anti-HCV antibody negative and HCV RNA positive at screening, the estimated date of infection was six weeks before enrolment, regardless of symptom status.

*HCV RNA Sequencing*

HCV RNA was extracted from EDTA plasma or ACD plasma using QIAamp viral extraction mini kit (#52906, QIAGEN, Limburg, NL). Reverse transcription and polymerase chain reaction (PCR) amplification of a region of the HCV genome encoding Core, Envelope-1 (E1) and the beginning of Envelope-2 (E2) was performed to generate a 1,404 base pair (bp) amplicon (nucleotides 347-1750 in H77 reference sequence [GenBank ascension no. NC_004102]) using a method previously described [[5](#_ENREF_5)]. PCR amplicons were sequenced by Sanger sequencing and sequence chromatograms were processed using RECall: a fully automated sequence analysis pipeline [[6](#_ENREF_6)]. Subtypes were determined by constructing a subtyping tree using the panel of reference sequences classified by Smith et al [[7](#_ENREF_7)].

**References**

1. Matthews, G.V., et al., *Patterns and characteristics of hepatitis C transmission clusters among HIV-positive and HIV-negative individuals in the Australian trial in acute hepatitis C.* Clin Infect Dis, 2011. **52**(6): p. 803-11.

2. Bradshaw, D., et al., *A comparison of seminal hepatitis C virus (HCV) RNA levels during recent and chronic HCV infection in HIV-infected and HIV-uninfected individuals.* J Infect Dis, 2015. **211**(5): p. 736-43.

3. Martinello, M., et al., *Short duration response-guided treatment is effective for most individuals with recent hepatitis C infection: the ATAHC II and DARE-C I studies.* Antivir Ther, 2016. **21**(5): p. 465.

4. Martinello, M., et al., *Sofosbuvir and ribavirin for 6 weeks is not effective among people with recent hepatitis C virus infection: The DARE-C II study.* Hepatology, 2016. **64**(6): p. 1911-1921.

5. Lamoury, F.M., et al., *The Influence of Hepatitis C Virus Genetic Region on Phylogenetic Clustering Analysis.* PLoS One, 2015. **10**(7): p. e0131437.

6. Woods, C.K., et al., *Automating HIV Drug Resistance Genotyping with RECall, a Freely Accessible Sequence Analysis Tool.* Journal of Clinical Microbiology, 2012. **50**(6): p. 1936-1942.

7. Smith, D.B., et al., *Expanded classification of hepatitis C virus into 7 genotypes and 67 subtypes: updated criteria and genotype assignment web resource.* Hepatology, 2014. **59**(1): p. 318-27.

**Supplementary figure legends**

**Supplementary Figure 1.** Maximum likelihood phylogenetic trees inferred from available hepatitis C virus (HCV) Core-E2 sequence from five studies of recent HCV infection in Australia and New Zealand recruited between 2004 and 2015. Trees were inferred separately for each genotype, including references sequences obtained from online databases (black tips). Any clade that contained only reference sequences was collapsed to reduce tree length (represented as a black triangle). Scale bars indicate nucleotide substitutions per site. Study sequences represented as tips in the trees are colour coded according to their clustering status (blue; unlinked, yellow; in a pair, purple; in a cluster). Pairs and clusters are numbered corresponding to Figure 2 in the manuscript.


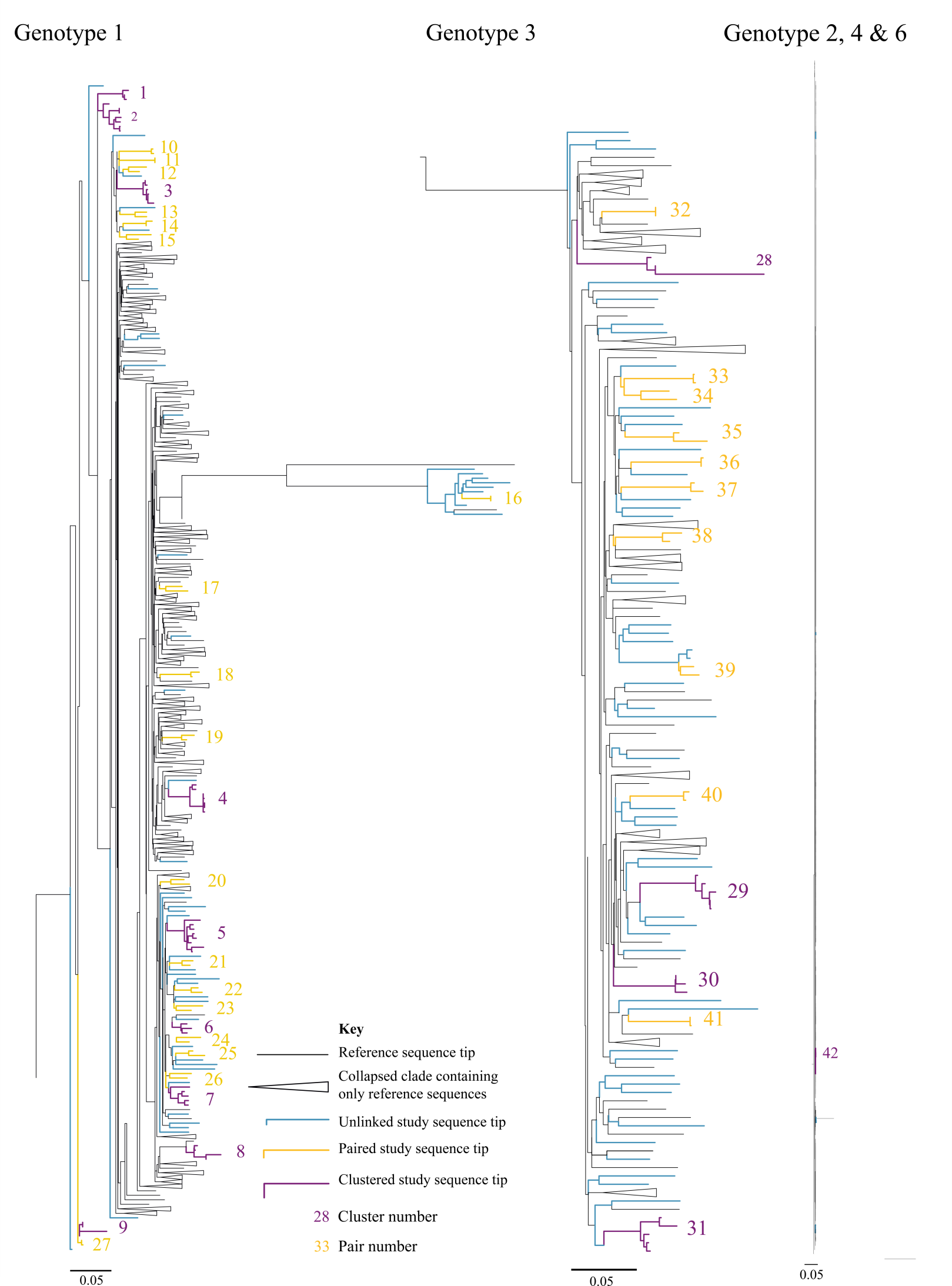
**Supplementary Figure 1**

| **Supplementary Table 1.** Multivariate logistic regression of factors associated with phylogenetic clustering among hepatitis C virus (HCV) Core-E2 sequences (at 5% genetic distance threshold) among participants from five studies of recent HCV infection in Australia and New Zealand recruited between 2004 and 2015. | | | | | | | | | | | | |
| --- | --- | --- | --- | --- | --- | --- | --- | --- | --- | --- | --- | --- |
| Characteristic | Overall | Individual | Clustered | Membership in cluster n ≥ 2 | | | | | | | | |
| Total n (%) | (n=237) | (n=129) | (n=108) | Unadjusted | | | Adjusted for HIV | | | Adjusted for HCV acquisition | | |
|  |  |  |  | Odds ratio | 95% CI | P | Odds ratio | 95% CI | P | Odds ratio | 95% CI | P |
| Age |  |  |  |  |  |  |  |  |  |  |  |  |
| Under 45 | 83 (35%) | 43 (33%) | 40 (37%) | Ref | - | - | Ref | - | - | Ref | - | - |
| Over 45 | 154 (65%) | 86 (67%) | 68 (63%) | 0.85 | 0.50, 1.45 | 0.552 | 1.15 | 0.58, 2.28 | 0.693 | 1.13 | 0.57, 2.26 | 0.720 |
| Gender |  |  |  |  |  |  |  |  |  |  |  |  |
| Female | 37 (16%) | 27 (21%) | 10 (9%) | Ref | - | - | Ref | - | - | Ref | - | - |
| Male | 187 (79%) | 91 (70%) | 96 (89%) | 2.85 | 1.31, 6.21 | 0.009 | 1.39 | 0.56, 3.48 | 0.475 | 1.60 | 0.66, 3.88 | 0.295 |
| Other^^^ | 13 (5%) | 11 (9%) | 2 (2%) |  |  |  |  |  |  |  |  |  |
| City |  |  |  |  |  |  |  |  |  |  |  |  |
| Other^#^ | 40 (17%) | 28 (21%) | 12 (11%) | Ref | - | - | Ref | - | - | Ref | - | - |
| Sydney | 109 (46%) | 57 (44%) | 52 (48%) | 2.13 | 0.98, 4.61 | 0.056 | 1.28 | 0.51, 3.20 | 0.597 | 1.48 | 0.61, 3.61 | 0.390 |
| Melbourne | 88 (37%) | 44 (34%) | 44 (41%) | 2.33 | 1.05, 5.17 | 0.037 | 1.77 | 0.75, 4.19 | 0.191 | 1.95 | 0.83, 4.56 | 0.123 |
| HIV infection |  |  |  |  |  |  |  |  |  |  |  |  |
| Negative | 114 (48%) | 80 (62%) | 34 (31%) | Ref | - | - | Ref | - | - | NI | NI | NI |
| Positive | 123 (52%) | 49 (38%) | 74 (69%) | 3.55 | 2.07, 6.09 | <0.001 | **2.30** | **1.07, 4.94** | **0.032** | NI | NI | NI |
| Acquisition of HCV^*^ |  |  |  |  |  |  |  |  |  |  |  |  |
| IDU | 121 (51%) | 74 (57%) | 47 (44%) | Ref | - | - | NI | NI | NI | Ref | - | - |
| Sexual | 97 (41%) | 39 (30%) | 58 (54%) | 2.34 | 1.36, 4.04 | 0.002 | NI | NI | NI | 2.14 | 0.94, 4.89 | 0.068 |
| Other | 19 (8%) | 16 (13%) | 3 (2%) | 0.30 | 0.08, 1.07 | 0.063 | NI | NI | NI | 0.35 | 0.03, 3.58 | 0.378 |
| HCV Genotype |  |  |  |  |  |  |  |  |  |  |  |  |
| 1a | 131 (55%) | 86 (67%) | 45 (42%) | Ref | - | - | Ref | - | - | Ref | - | - |
| 3a | 89 (38%) | 38 (29%) | 51 (47%) | 2.56 | 1.47, 4.46 | 0.001 | 1.86 | 0.97, 3.57 | 0.061 | **2.09** | **1.11, 3.95** | **0.023** |
| Other | 17 (7%) | 5 (4%) | 12 (11%) | 4.59 | 1.52, 13.83 | 0.007 | 2.95 | 0.90, 9.66 | 0.074 | **3.98** | **1.21, 13.02** | **0.022** |
| Injection drug use |  |  |  |  |  |  |  |  |  |  |  |  |
| Injected ever, but  not recently^†^ | 78 (33%) | 46 (36%) | 32 (30%) | Ref | - | - | Ref | - | - | Ref | - | - |
| Injected recently^†^ | 89 (38%) | 48 (37%) | 41 (38%) | 1.23 | 0.66, 2.70 | 0.512 | 1.22 | 0.61, 2.43 | 0.569 | 1.35 | 0.66, 2.75 | 0.412 |
| Never injected | 57 (24%) | 25 (19%) | 32 (30%) | 1.84 | 0.92, 3.67 | 0.084 | 1.15 | 0.0.53, 2.55 | 0.709 | 1.06 | 0.43, 2.60 | 0.904 |
| Unknown | 13 (5%) | 10 (8%) | 3 (2%) | 0.43 | 0.11, 1.69 | 0.228 | 0.66 | 0.08, 5.25 | 0.697 | 0.85 | 0.10, 7.12 | 0.884 |
| Percentages indicate column percentages. Abbreviations: hepatitis C virus (HCV); Injecting Drug Use (IDU); Confidence Intervals (CI), Not Included (NI); Reference (Ref).  ^^^Other includes one transgender subject, and 12 subjects for which this variable was unknown  ^#^ Adelaide, Newcastle, Auckland, Brisbane or Perth  *Acquisition was determined by the clinician according to reported risk factors  ^†^Within last 6 months prior to sample date | | | | | | | | | | | | |

| **Supplementary Table 2.** Multivariate logistic regression of factors associated with phylogenetic clustering among hepatitis C virus (HCV) Core-E2 sequences (at 5% genetic distance threshold) stratified among **HCV mono-infected** participants from five studies of recent HCV infection in Australia and New Zealand recruited between 2004 and 2015. | | | | | | | | | |
| --- | --- | --- | --- | --- | --- | --- | --- | --- | --- |
| Characteristic | Overall | Unclustered | Clustered | Membership in cluster n ≥ 2 | | | | | |
| Total n (%) | (n=114) | (n=80) | (n=34) | Unadjusted | | | Adjusted (full model) | | |
|  |  |  |  | Odds ratio | 95% CI | P | Odds ratio | 95% CI | P |
| Age |  |  |  |  |  |  |  |  |  |
| Under 45 | 25 (22%) | 17 (21%) | 8 (24%) | Ref | - | - | Ref | - | - |
| Over 45 | 89 (78%) | 63 (79%) | 26 (76%) | 0.87 | 0.34, 2.28 | 0.788 | 0.92 | 0.18, 4.62 | 0.922 |
| Gender |  |  |  |  |  |  |  |  |  |
| Female | 37 (32%) | 27 (34%) | 10 (29%) | Ref | - | - | Ref | - | - |
| Male | 64 (56%) | 42 (53%) | 22 (65%) | 1.41 | 0.58, 3.44 | 0.445 | 1.37 | 0.47, 3.95 | 0.562 |
| Other^^^ | 13 (12%) | 11 (13%) | 2 (6%) | 0.54 | 0.10, 2.91 | 0.473 | ^∞^ | ^∞^ | ^∞^ |
| City |  |  |  |  |  |  |  |  |  |
| Melbourne | 45 (39%) | 33 (41%) | 12 (35%) | Ref | - | - | Ref | - | - |
| Sydney | 35 (31%) | 22 (28%) | 13 (38%) | 1.63 | 0.63, 4.21 | 0.318 | 1.83 | 0.56, 6.04 | 0.319 |
| Other^#^ | 34 (30%) | 25 (31%) | 9 (27%) | 0.99 | 0.36, 2.71 | 0.984 | 0.60 | 0.19, 1.88 | 0.379 |
| Acquisition of HCV^*^ |  |  |  |  |  |  |  |  |  |
| Injecting drug use | 91 (80%) | 63 (79%) | 28 (82%) | Ref | - | - | ^§^ | ^§^ | ^§^ |
| Sexual | 4 (3%) | 1 (1%) | 3 (4%) | 6.76 | 0.67, 67.77 | 0.105 | ^∞^ | ^∞^ | ^∞^ |
| Other | 19 (17%) | 16 (20%) | 3 (4%) | 0.42 | 0.11, 1.57 | 0.197 | ^∞^ | ^∞^ | ^∞^ |
| HCV Genotype |  |  |  |  |  |  |  |  |  |
| 1a | 86 (75%) | 68 (85%) | 18 (53%) | Ref | - | - | Ref | - | - |
| 3a | 25 (22%) | 12 (15%) | 13 (40%) | 4.09 | 1.60, 10.49 | 0.003 | **4.35** | **1.42, 13.30** | **0.010** |
| Other^∞^ | 3 (3%) | 0 (0%) | 3 (9%) | ^∞^ | ^∞^ | ^∞^ | ^∞^ | ^∞^ | ^∞^ |
| Injection drug use |  |  |  |  |  |  |  |  |  |
| Injected ever, but not  recently^†^ | 41 (36%) | 31 (39%) | 10 (29%) | Ref | - | - | Ref | - | - |
| Injected recently^†§^ | 53 (46%) | 35 (44%) | 18 (53%) | 1.59 | 0.64, 3.97 | 0.316 | ^§^ | ^§^ | ^§^ |
| Never injected | 10 (9%) | 6 (8%) | 4 (12%) | 2.07 | 0.48, 8.83 | 0.327 | ^∞^ | ^∞^ | ^∞^ |
| Unknown | 10 (9%) | 8 (9%) | 2 (6%) | 0.78 | 0.14, 4.27 | 0.770 | ^∞^ | ^∞^ | ^∞^ |
| Percentages indicate column percentages. Abbreviations: Confidence Intervals (CI), Reference (Ref).  ^^^Other includes one transgender subject, and 12 subjects for which this variable was unknown  ^#^ Adelaide, Newcastle, Auckland, Brisbane or Perth  *Acquisition was determined by the clinician according to reported risk factors  ^∞^Variable omitted from logistic regression due to insufficient power  ^†^Within last 6 months prior to sample date  ^§^Variable omitted due to collinearity | | | | | | | | | |

| **Supplementary Table 3.** Multivariate logistic regression of factors associated with phylogenetic clustering of hepatitis C virus (HCV) Core-E2 sequences (at 5% genetic distance threshold) among **HIV/HCV co-infected** participants from five studies of recent HCV infection in Australia and New Zealand recruited between 2004 and 2015. | | | | | | | | | |
| --- | --- | --- | --- | --- | --- | --- | --- | --- | --- |
| Characteristic | Overall | Unclustered | Clustered | Membership in cluster n ≥ 2 | | | | | |
| Total n (%) | (n=123) | (n=49) | (n=74) | Unadjusted | | | Adjusted (Full model) | | |
|  |  |  |  | Odds ratio | 95% CI | P | Odds ratio | 95% CI | P |
| Age |  |  |  |  |  |  |  |  |  |
| Under 45 | 58 (%) | 26 (%) | 32 (%) | Ref | - | - | Ref | - | - |
| Over 45 | 65 (%) | 23 (%) | 42 (%) | 1.48 | 0.72, 3.06 | 0.286 | 1.19 | 0.53, 2.65 | 0.665 |
| City |  |  |  |  |  |  |  |  |  |
| Sydney | 74 (60%) | 35 (71%) | 39 (53%) | Ref | - | - | Ref | - | - |
| Melbourne | 43 (35%) | 11 (22%) | 32 (43%) | 1.11 | 0.21, 5.88 | 0.899 | 2.89 | 0.49, 17.16 | 0.242 |
| Other^#^ | 6 (5%) | 3 (6%) | 3 (4%) | 2.91 | 0.51, 16.59 | 0.229 | ^∞^ | ^∞^ | ^∞^ |
| Acquisition of HCV^*^ |  |  |  |  |  |  |  |  |  |
| Sexual | 93 (%) | 38 (%) | 55 (%) | Ref | - | - | Ref | - | - |
| Injecting drug use | 30 (%) | 11 (%) | 19 (%) | 1.19 | 0.51, 2.79 | 0.683 | 1.87 | 0.53, 6.57 | 0.326 |
| HCV Genotype |  |  |  |  |  |  |  |  |  |
| 1a | 45 (37%) | 18 (37%) | 27 (37%) | Ref | - | - | Ref | - | - |
| 3a | 64 (52%) | 26 (53%) | 38 (51%) | 0.97 | 0.45, 2.12 | 0.948 | 0.81 | 0.33, 2.01 | 0.654 |
| Other | 14 (11%) | 5 (10%) | 9 (12%) | 1.20 | 0.37, 4.10 | 0.774 | 1.08 | 0.27, 4.39 | 0.912 |
| Injection drug use |  |  |  |  |  |  |  |  |  |
| Injected ever, but not  recently | 37 (30%) | 15 (31%) | 22 (30%) | Ref | - | - | Ref | - | - |
| Injected recently^†^ | 36 (29%) | 13 (27%) | 23 (31%) | 1.21 | 0.47, 3.10 | 0.697 | 1.19 | 0.40, 3.58 | 0.754 |
| Never injected | 47 (38%) | 19 (39%) | 28 (38%) | 1.00 | 0.42, 2.42 | 0.991 | 1.20 | 0.45, 3.24 | 0.710 |
| Unknown | 3 (3%) | 2 (3%) | 1 (1%) | 0.34 | 0.03, 4.11 | 0.397 | 0.51 | 0.04, 7.13 | 0.615 |
| Percentages indicate column percentages. Abbreviations: Confidence Intervals (CI), Reference (Ref).  ^#^ Adelaide, Newcastle, Auckland, Brisbane or Perth  ^∞^Variable omitted from logistic regression due to insufficient power  *Acquisition was determined by the clinician according to reported risk factors  ^†^Within last 6 months prior to sample date | | | | | | | | | |

| **Supplementary Table 4.** Comparison of fit statistics for Latent Class Analysis models built with 1 to 8 classes for participants from five studies of recent HCV infection in Australia and New Zealand recruited between 2004 and 2015. | | | | | | | | | | | |
| --- | --- | --- | --- | --- | --- | --- | --- | --- | --- | --- | --- |
| Number of classes | log-likelihood | G-squared | AIC | BIC | cAIC | aBIC | Entropy | Degrees-of-freedom | mCAR-log-likelihood | mCAR-G-squared | mCAR-degrees-of-freedom |
| 1 | -949.09 | 437.23 | 459.23 | 497.38 | 508.38 | 462.51 | 1 | 156 | -730.47 | 87.24 | 109 |
| 2 | -796.72 | 132.51 | 178.51 | 258.27 | 281.27 | 185.37 | 0.93 | 144 | -730.47 | 87.24 | 109 |
| 3 | -763.42 | 65.91 | 135.91 | 257.29 | 292.29 | 146.35 | 0.88 | 132 | -730.47 | 87.24 | 109 |
| 4 | -752.62 | 44.3 | 138.3 | 301.3 | 348.3 | 152.33 | 0.87 | 120 | -730.47 | 87.24 | 109 |
| 5 | -743.05 | 25.15 | 143.15 | 347.77 | 406.77 | 160.76 | 0.89 | 108 | -730.47 | 87.24 | 109 |
| 6 | -737.75 | 14.56 | 156.56 | 402.79 | 473.79 | 177.75 | 0.89 | 96 | -730.47 | 87.24 | 109 |
| 7 | -734.93 | 8.92 | 174.92 | 462.77 | 545.77 | 199.69 | 0.83 | 84 | -730.47 | 87.24 | 109 |
| 8 | -733.58 | 6.21 | 196.21 | 525.68 | 620.68 | 224.56 | 0.8 | 72 | -730.47 | 87.24 | 109 |
| Abbreviations: The Bayesian Information Criterion (BIC), Akaike Information Criterion (AIC), adjusted BIC (aBIC), adjusted AIC (aAIC), Missing Completely at Random (mCAR). | | | | | | | | | | | |
